# A pH-Responsive Zwitterionic Polyurethane Prodrug as Drug Delivery System for Enhanced Cancer Therapy

**DOI:** 10.3390/molecules26175274

**Published:** 2021-08-31

**Authors:** Qian He, Rui Yan, Wanting Hou, Haibo Wang, Yali Tian

**Affiliations:** 1 Department of Emergency, West China Hospital, Sichuan University, Guoxue Alley No. 37, Chengdu 610041, China; heqian2020@wchscu.cn (Q.H.); hwt9229@126.com (W.H.); 2 College of Biomass Science and Engineering, Sichuan University, Chengdu 610041, China; bigfacecat2021@126.com (R.Y.); whb6985@scu.edu.cn (H.W.); 3 West China School of Nursing, West China Hospital, Sichuan University, Guoxue Alley No. 37, Chengdu 610041, China

**Keywords:** pH-responsive polyurethane, Adriamycin, zwitterionic, antitumor

## Abstract

Numerous nanocarriers with excellent biocompatibilities have been used to improve cancer therapy. However, nonspecific protein adsorption of nanocarriers may block the modified nanoparticles in tumor cells, which would lead to inefficient cellular internalization. To address this issue, pH-responsive polyurethane prodrug micelles with a zwitterionic segment were designed and prepared. The micelle consisted of a zwitterionic segment as the hydrophilic shell and the drug Adriamycin (DOX) as the hydrophobic inner core. As a pH-responsive antitumor drug delivery system, the prodrug micelles showed high stability in a physiological environment and continuously released the drug under acidic conditions. In addition, the pure polyurethane carrier was demonstrated to be virtually non-cytotoxic by cytotoxicity studies, while the prodrug micelles were more efficient in killing tumor cells compared to PEG-PLGA@DOX. Furthermore, the DOX cellular uptake efficiency of prodrug micelles was proved to be obviously higher than the control group by both flow cytometry and fluorescence microscopy. This is mainly due to the modification of a zwitterionic segment with PU. The simple design of zwitterionic prodrug micelles provides a new strategy for designing novel antitumor drug delivery systems with enhanced cellular uptake rates.

## 1. Introduction

Nowadays, the topic of health has attracted a lot of attention. As reported by the World Health Organization (WHO), cancer kills nearly 18 million people per year and has become a major threat to human health [1]. Currently, most conventional chemotherapeutic agents (e.g., Adriamycin (DOX), methotrexate, and camptothecin) have serious disadvantages that need to be addressed, such as low selectivity, low water solubility, substantial side effects, and short circulation time in the body [2,3]. To overcome these problems, scientists have developed a range of nanocarriers, such as nanoparticles, liposomes, nanogels and micelles through nanotechnology [4,5,6,7,8], such as nanocarrier-based polymer prodrugs. The hydrophobic segments of the polymer are linked to the hydrophobic drug by breakable chemical bonds to form the core of the micelle, and the hydrophilic segments form the micelle shell, providing good stability to the drug and prolonging the circulation time in vivo. Moreover, polymeric prodrug micelles with a particle size of less than 200 nm can aggregate at the tumor site selectively through the EPR effect [9,10]. All these advantages make polymeric micellar prodrugs a good potential drug delivery system.

Recently, the great nonfouling capability of zwitterionic polymers has been considered as the alternative of PEG [11,12,13,14,15,16,17,18,19,20,21,22]. Jiang’s group [23,24,25] has prepared several polymers (such as PCBMA, PSBMA and PMPC), which exhibited excellent stability and antifouling properties in vivo. Meanwhile, it has been reported that the cellular uptake efficiency of micelles would be increased by the modification of a zwitterionic segment in tumor tissues [26,27]. The main reason is the difference in pH between normal tissue cells (pH = 7.4) and tumor tissue (acidic). In the acidic environment, the zwitterionic groups undergo protonation. The ζ-potential would vary from slightly negatively charged in the normal tissue site to positively charged in the tumor tissue site. Therefore, the affinity between negatively charged tumor cell membranes and zwitterionic polymer micelles is enhanced under acidic conditions, leading to the increasing uptake of micelles by tumor cells.

Unfortunately, these backbones of the zwitterionic materials prepared by living polymerization or free radical polymerization cannot be eliminated through metabolism. In contrast, polyurethanes showed many advantages, including easy synthesis, excellent biocompatibility, biological inertness, and easy introduction of target molecules, which allowed them to be designed as stimulus-responsive polymeric structures and to be the most promising drug carriers for next-generation nanotherapies. 

**Scheme 1:**
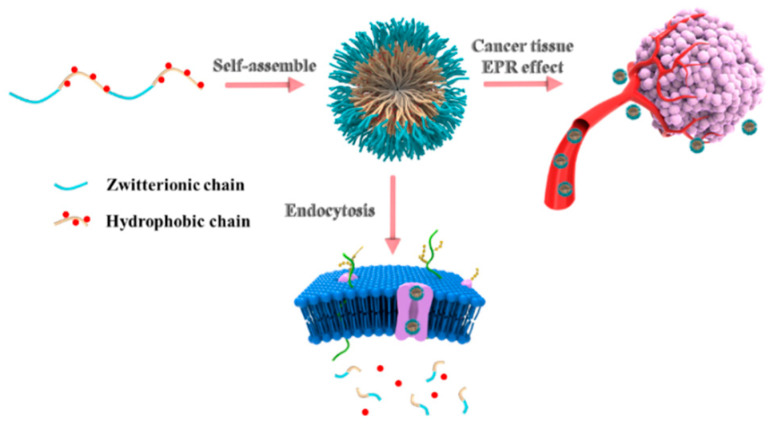
Illustration of pH-responsive prodrug self-assembly and in vivo drug release for enhanced cellular uptake.

Herein, in this paper, an amphiphilic copolymer with pH-responsive properties was constructed by zwitterionic monomer (dihydroxy carboxybetaine, DHCB), ethyl 2,6-diisocyanatohexanoate (LDI) and DOX via polyaddition reaction. As shown in Scheme 1, the amphiphilic polyurethane was polymerized by DHCB and LDI. Then, the resulting amphiphilic polyurethane was further amidated to react with DOX. The obtained product is supposed to self-assemble into prodrug micelles with excellent stability. Additionally, the micelles could be well dispersed in water and even in physiological solution. UV-visible absorption spectroscopy and dynamic light scattering (DLS) were also conducted to further characterize the properties of prodrug micelles. Furthermore, the drug release of the prodrug micelles was studied under different pH conditions, the internalization of the drug by cells was investigated by flow cytometry and the corresponding mechanisms were discussed.

## 2. Results and Discussion

### 2.1. The Characterization of Monomer and Polymer

**Figure 1:**
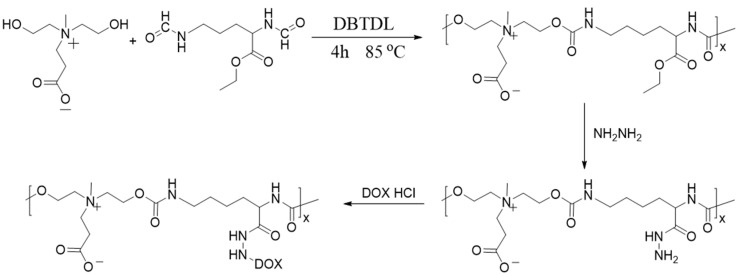
Detailed synthetic route of the PU-hyd-DOX.

**Figure 2:**
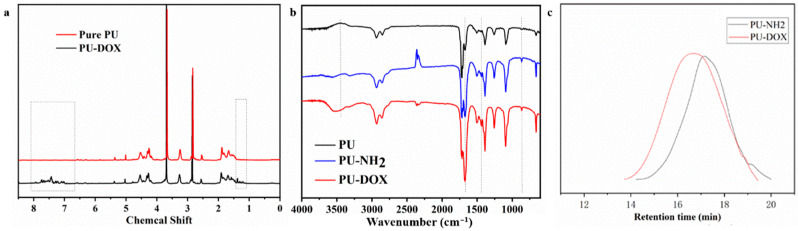
Structural characterization of polymers: (**a**) ^1^H NMR spectra of pure PU and PU-hyd-DOX; (**b**) FT-IR spectra of pure PU, PU-NH_2_ and PU-hyd-DOX; (**c**) The elution time of PU-NH_2_ and PU-hyd-DOX.

The detailed synthesis steps of zwitterionic PU prodrug with pH response are shown in Figure 1. Pure PU was prepared according to the previous method, and then the obtained PU was aminated by hydrazine hydrate. Subsequently, the aminated PU and DOX were dissolved together in a certain ratio. After a period of reaction, the PU prodrugs with pH corresponding functions were obtained by dialysis. Figure 2a showes the comparative NMR spectra of PU before and after accessing DOX. For pure PU, the characteristic peaks of methyl and methylene on DHCB appeared between 2.8 ppm and 5.1 ppm, while the methyl and methylene peaks on LDI appeared between 1.8 ppm and 2.0 ppm. The methyl and methylene groups on DHCB can be distinguished from LDI owing to the large chemical shift caused by the electron absorption of N^+^ on DHCB. Additionally, it was clearly seen that after accessing DOX, the peaks of double bonds and benzene rings appeared between 7 ppm and 8 ppm, and some methylene peaks also appeared between 1 ppm and 1.5 ppm. The structure of the polymers was further confirmed by the FT-IR spectra (Figure 2b). In the spectrum, a typical signal at 1698 cm^−1^ reveals the appearance of -NH-COO-. The aminated PU showed a stretching vibration peak at 1480 cm^−1^ for the C-N bonds. The presence of the characteristic benzene ring peak at 3050 cm^−1^ and the C=C double bond stretching vibration peak at 1650 cm^−1^ are further proof that DOX had been grafted to the PU chain. GPC was utilized to characterize the molecular weight of polyurethane; the retention time of PU-NH_2_ was shorter than the PU-hyd-DOX. The above results confirmed the successful synthesis of PU-hyd-DOX.

### 2.2. The Characterization of Micelles and In Vitro Release of PU-hyd-DOX

**Figure 3:**
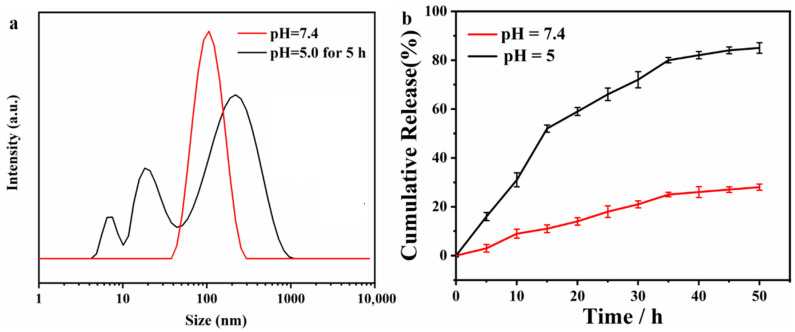
The characterization of micelles and in vitro release of PU-hyd-DOX: (**a**) the particle size of micelles in different conditions; (**b**) the drug release of PU-hyd-DOX in vitro.

The prodrug micelles consist of the hydrophobic drug segment and the hydrophilic zwitterionic segment, so that the PU chains can self-assemble into micelles in aqueous solution, with the drug as the core and the zwitterionic chain segment as the shell. To verify the structure, the particle size was tested by DLS, and the micelles were around 100 nm in size and monodisperse (Figure 3a). Moreover, the pH-sensitive ability was also characterized in the acidic solution. As shown in the Figure 3a, the particle size became scattered after being stored at pH = 5 for 5 h, indicating that the micelles collapsed under acidic conditions and the molecular chains broke. The results proved the excellent pH responsiveness of PU-hyd-DOX. Then, the in vitro release capability of the polymeric prodrug was characterized. It can be seen from Figure 3b that at pH = 7.4 the micelles showed a relatively stable state, with only 20% of the drug released after 50 h of placement. In contrast, the prodrug micelles exceeded 20% in just 10 h when placed at pH = 5 and took only 35 h to release more than 80% of the drug. The results showed that the prodrug micelles remain relatively stable in the normal human physiological environment and showed a rapid response to release in an acidic environment, i.e., at the tumor site.

### 2.3. Cytotoxicity Study

**Figure 4:**
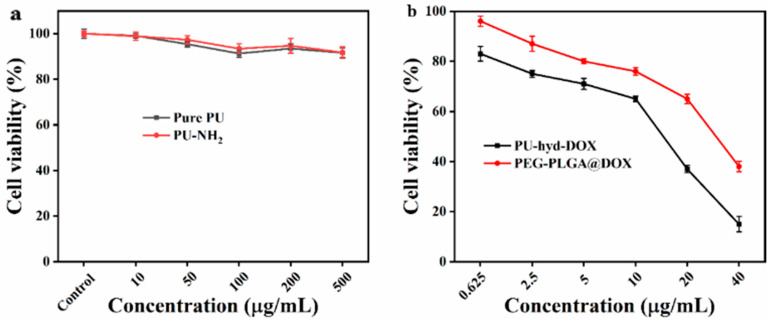
Cytotoxicity study of polymers and prodrug: (**a**) Pure PU and PU-NH_2_; (**b**) PEG-PLGA@DOX and PU-hyd-DOX.

The cck-8 method was used to determine the relative toxicity of pure and aminated PU to normal tissue cells. As can be seen from Figure 4, the cell viability was higher than 90% at different concentrations, indicating that the cell activity was not affected by the material. Therefore, the resulting zwitterionic PU has good cytocompatibility. The relative toxicity of DOX and PU-hyd-DOX pre-drug micelles to tumor cells was compared. The results showed that the prodrug micelles exhibited excellent tumor-killing activity against HepG2 (human hepatocellular carcinoma tumor cell line). It should be noted that the prodrug micelles showed a better inhibition rate compared to PEG-PLGA@DOX, which contrasted with the results of most prodrug micelle studies. Therefore, the reasons for their enhanced cell-killing should be investigated.

### 2.4. Cellular Uptake

**Figure 5:**
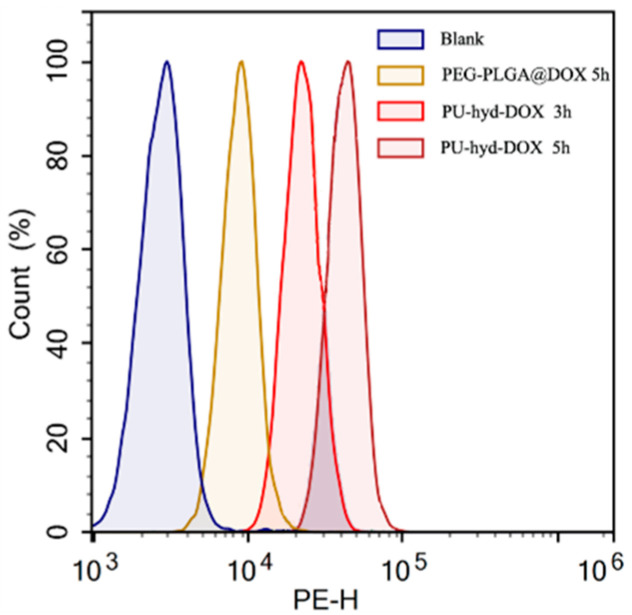
Flow cytometry analysis of cellular uptake in HepG2 cells at pH 7.4 and 37 °C when treated by PEG-PLGA@DOX and PU-hyd-DOX micelles after different incubation times.

To study the mechanism of enhanced cytotoxicity of the polymer prodrug micelles, the cellular uptake of PEG-PLGA@DOX and PU-hyd-DOX micelles at different times was studied using both flow cytometry and fluorescence microscopy. The results from flow cytometry (Figure 5) showed that PEG-PLGA@DOX was consistently lower than the cellular uptake rate of the prodrug micelles. Additionally, fluorescence microscopy further supported this view. 

**Figure 6:**
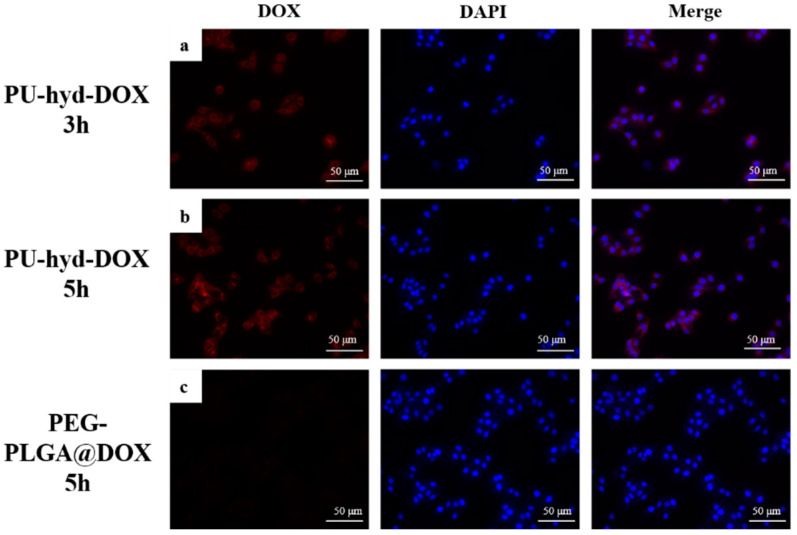
The fluorescent microscopy images of the different incubation times of HepG2 cells with PEG-PLGA@DOX and PU-hyd-DOX micelles at pH 7.4; (**a**) PU-hyd-DOX 3 h (**b**) PU-hyd-DOX 5 h (**c**) PEG-PLGA@DOX 5 h.

Figure 6a,b show the prodrug micelles co-cultured with cells for 3 h and 5 h, respectively, and Figure 6c shows PEG-PLGA@DOX co-cultured with cells for 5 h. The results show that the fluorescence signal of the prodrug micelles was higher than that of PEG-PLGA@DOX at the same exposure time, indicating a more efficient cellular uptake of the prodrug micelles. The results were consistent with flow cytometry. This result was mainly attributed to the modification of the prodrug micelles with a zwitterionic segment to improve the uptake efficiency of the micelles by the cells.

## 3. Materials and Methods

### 3.1. Materials

Doxorubicin hydrochloride (DOX·HCl), hydrazine hydrate and ethyl 2,6-diisocyanatohexanoate (LDI) were obtained from J&K Chemical Co., Ltd. (Shanghai, China). Dibutyltin dilaurate (DBTBL) was obtained from KeLong Chemical Co., Ltd. (Liaoning, China). All other solvents and reagents were purchased from KeLong Chemical Co., Ltd. Deionized water was homemade and used throughout the experiments.

### 3.2. Instrumentations

The ^1^H-NMR spectra were collected by Bruker AV III HD (400 MHz; Bremen, Germany). A Nicolet 560 spectrum scanner (Thermo Scientific, Waltham, MA, USA) in diffuse reflectance mode was used to collected the Fourier transform infrared (FT-IR) spectra. The size and distribution of the micelles in water was measured by the Zetasizer Nano S90 (Malvern Instruments, Malvern, UK). A gel permeation chromatography (GPC) was exploited to characterize the molecular weights and polydispersities of the polymers. using DMF as the eluent. Fluorescence intensity of DOX was analyzed for cellular internalization using fluorescence microscopy and flow cytometry (BD FACSCalibur). Additionally, cell viability was recorded using a SpectraMax M2 instrument after the CCK-8 assay. Every experiment was performed for three times, and the average was used in quantitative analysis.

### 3.3. Synthesis of DHCB

According to the previous research [28], the DHCB was synthesized by three steps. Firstly, diethanolamine and tert-butyl acrylate reacted for 48 h at room temperature. Then, the obtained products reacted with iodomethane. Finally, a mixture of DCM and TFA (*v*/*v* = 1/1) was used to produce the DHCB via stirring for 3 h. A 400 MHz spectrometer was used to confirm the chemical structure in methol-D4. 1H NMR (400 MHz, 500 μL CD_3_OD), δ (ppm) = 4.01 (m, 4H), 3.82 (t, *J* = 15 Hz, 2H), 3.64 (m, 4H), 3.30 (s, 3H), 3.09 (t, *J* = 15 Hz, 2H).

### 3.4. Synthesis of PU Prodrug (PU-hyd-DOX)

According to the standard procedure, the polyurethane was synthesized by the following steps. Firstly, the trace water was removed from all the reagents. Then, DHCB (0.5 g) and LDI (0.897 g) were placed in a 50 mL flask, and DMF (5 mL) as the solution was used to dissolve them, and catalyst was injected. The temperature of reaction was maintained at 85 °C under N_2_ for 4 h. Hydrazine hydrate was then added to the solution and the ethyl group on the main chain was substituted by a substitution reaction to give the aminated PU. A cold anhydrous ether was used to precipitate the reaction mixture. Immediately afterwards, the aminated PU was dissolved with DOX and stirred to obtain a PU with a hydrazone bond structure (pH sensitive). The chemical structures were confirmed by the ^1^H NMR and infrared spectrum.

### 3.5. Preparation of Polyurethane Prodrug Micelles

The polymer micelles were prepared by the dialysis method. Briefly, 10 mL distilled water was added into the mixture of polymer (10 mL) and DMSO (1.5 mL) under vigorous stirring. Then, a dialysis membrane (MWCO = 3.5 kDa) was used to dialyze the mixture against deionized water to remove DMSO. The 0.45 μm pore-sized microporous membrane was utilized to filter the solution before detection. Additionally, the micelle behavior of PU-hyd-DOX micelles was analyzed by DLS.

### 3.6. The Stimuli-Response Behaviour and Vitro Drug Release 

The zwitterionic shell was expected to endow excellent physiological stability to the prodrug micelles. Dynamic light scattering (Malvern instruments) was used to determine the dimensions of micelles. The pH-responsive drug release behavior in vitro was evaluated by the dialysis method. Briefly, PU-hyd-DOX micelles were suspended in a dialysis bag (MWCO = 3.5 kDa) at different buffer solutions (pH = 7.4 or 5.0) with continuous stirring at 37 °C. Then, 1 mL release medium of the sample was detected the accumulative amount of the release of DOX by the UV spectroscopy. The experiment was conducted three times.

### 3.7. Cell Culture

DMEM with 10% heat inactivated PBS, 1% penicillin, and 1% of streptomycin was used to culture the HepG2 cells. The culture condition of the cells was maintained at 37 °C and 5% CO_2_ atmosphere. Every group of the culture medium was changed every 2 days to maintain the exponential growth of the cells.

### 3.8. Cell Cytotoxicity Assay

The cytotoxicity of pure PU micelles and PU-hyd-DOX micelles was determined by CCK-8 with HepG2 cells, respectively. 96-well plates were used to seed cells (the volume of each well was 100 μL). After the cells were attached to the plate for 24 h, serial concentrations of the sample’s micelles solution (0.625, 1.25, 2.5, 5, 10, 20, 40 μg/mL) were conducted to culture the cells for 48 h. Then, PBS solution was used to wash away the free materials. The cells’ survival rate was measured by the CCK-8 solution. Briefly, 10 μL of CCK-8 in 100 μL of DMEM cell culture medium was added to each well, and the cells were incubated for another 4 h at 37 °C. Then, the cell incubation plates were analyzed by a microplate reader (Thermo Multiskan GO). Every experiment was conducted three times, and the results in this work are presented as the mean ± standard deviation (SD).

### 3.9. Cellular Uptake Assay 

The cellular uptake behavior of micelles was measured by flow cytometry and fluorescence microscopy. For flow cytometry measurements, 12-well plates were used to seed the HepG2 cells, and the cells were incubated for 24 h. Then, medium was replaced with pH-modified (7.4 and 5.0) serum-free DMEM including PEG-PLGA@DOX (a commercial drug-carrier encapsulated DOX) and PU-hyd-DOX micelles, respectively. The final concentration of DOX in medium was 1.8 μg/mL. The different pH medium was removed after 20 h. The plates were washed with PBS several times. Cells were collected by using 200 μL trypsin per well. The cells were suspended in 1 mL PBS to detect the internalization level by flow cytometer. The mean fluorescence intensity ± standard deviation of DOX was obtained and the results were analyzed using Flowjo 7.6 analysis program. For fluorescence microscopy measurements, HepG2 cells were seeded in 96-well plates and incubated for 24 h. The medium was replaced with pH 7.4 serum-free DMEM medium containing PEG-PLGA@DOX and PU-hyd-DOX micelles. The final concentration of DOX in medium was 1.8 μg/mL. The medium was removed after 20 h. The plates were washed with cold PBS. Cell nuclei were stained using 5 μg/mL DAPI to obtain fluorescent images.

## 4. Conclusions

In summary, a zwitterionic pH-responsive prodrug micelle consisting of a hydrophilic zwitterion segment and a hydrophobic drug DOX was prepared by an extremely simple method. The prodrug micelles exhibited excellent drug release efficiency at pH = 5 and showed excellent stability at pH = 7.4. In addition, cytotoxicity assays demonstrated good biocompatibility of the polymeric material and enhanced cytotoxicity of the prodrug micelles compared to the PEG-PLGA@DOX. This was attributed to the effect of the zwitterionic segment in the prodrug micelles, which increased the uptake of DOX by the cells. Thus, prodrug micelles are expected to be applied to overcome the cellular uptake challenges for free DOX, providing a new idea for future research on drug delivery systems.
